# Nasal Drug Delivery in Traditional Persian Medicine

**DOI:** 10.17795/jjnpp-9990

**Published:** 2013-07-16

**Authors:** Mohammad Mehdi Zarshenas, Arman Zargaran, Johannes Müller, Abdolali Mohagheghzadeh

**Affiliations:** 1 Student Research Committee, Shiraz University of Medical Sciences, Shiraz, IR Iran; 2 Pharmaceutical Sciences Research Center, Shiraz University of Medical Sciences, Shiraz, IR Iran; 3 Department of Traditional Pharmacy, School of Pharmacy, Shiraz University of Medical Sciences, Shiraz, IR Iran; 4 Research Office for the History of Persian Medicine, Shiraz University of Medical Sciences, Shiraz, IR Iran; 5 University of Marburg, Institute for the History of Pharmacy, Germany

**Keywords:** Herbal Medicine, Dosage Form, Medicine, Traditional

## Abstract

**Background:**

Over one hundred different pharmaceutical dosage forms have been recorded in literatures of Traditional Persian Medicine among which nasal forms are considerable.

**Objectives:**

This study designed to derive the most often applied nasal dosage forms together with those brief clinical administrations.

**Materials and Methods:**

In the current study remaining pharmaceutical manuscripts of Persia during 9th to 18th century AD have been studied and different dosage forms related to nasal application of herbal medicines and their therapeutic effects were derived.

**Results:**

By searching through pharmaceutical manuscripts of medieval Persia, different nasal dosage forms involving eleven types related to three main groups are found. These types could be derived from powder, solution or liquid and gaseous forms. Gaseous form were classified into fumigation (*Bakhoor*), vapor bath (*Enkebab*), inhalation (*Lakhlakheh*), aroma agents (*Ghalieh*) and olfaction or smell (*Shomoom*). Nasal solutions were as drops (*Ghatoor*), nasal snuffing drops (*Saoot*) and liquid snuff formulations (*Noshoogh*). Powders were as nasal insufflation or snorting agents (*Nofookh*) and errhine or sternutator medicine (*Otoos*). Nasal forms were not applied only for local purposes. Rather systemic disorders and specially CNS complications were said to be a target for these dosage forms.

**Discussion:**

While this novel type of drug delivery is known as a suitable substitute for oral and parenteral administration, it was well accepted and extensively mentioned in Persian medical and pharmaceutical manuscripts and other traditional systems of medicine as well. Accordingly, medieval pharmaceutical standpoints on nasal dosage forms could still be an interesting subject of study. Therefore, the current work can briefly show the pharmaceutical knowledge on nasal formulations in medieval Persia and clarify a part of history of traditional Persian pharmacy.

## 1. Background

The history of drug therapy goes back to long years ago when human began to treat the diseases using natural medicines (1). Since then, preparation of animal, herbal and mineral medicaments have been practiced to produce a therapeutic effect (2). What is accepted in the world today is that various medicines in different dosage forms and via specific route of administration are to be applied for related therapeutic purposes (3, 4). By going through the history of Persia, many documents involving information of medical and pharmaceutical sciences consist of compounding and preparing aspects of medicinal formulations would be found (5). During the medieval period Persian practitioners meticulously gathered the achieved experiences of other civilizations and combined the received data with their own findings and experiments (6, 7). Pharmaceutical manuscripts of Persian scholars involved many considerable aspects and contents of therapeutic dosage forms and treatment strategies. Over 100 dosage forms have been mentioned in pharmaceutical literatures of Traditional Persian Medicine (TPM) where nasal application involving related dosage forms is one of the most often mentioned routs of administration (8, 9). 

This approach which is known as a suitable substitute for oral and parenteral administration, (10) was significantly practiced from ancient era (11). For several years, medicaments have been applied nasally for their local pharmacological effects on the mucosa as well as systemic approaches (12). This route of drug delivery was considered by traditional systems of medicine such as Unani, Ayurvedic as well as Persian (13, 14). It was extensively considered by medieval Persian physicians and was presented as an important route of drug administration especially for neurologic disorders (14). A number of recorded nasal remedies can be found in Persian manuscripts. Therefore, this paper briefly discussed various types of nasal application along with their considerations in medieval Persia in order to clarify the pharmaceutical knowledge on nasal drug application during the medieval Persian period as part of the history of pharmacy. 

## 2. Objectives

This study designed to derive the most often applied nasal dosage forms together with those brief clinical administrations. 

## 3. Materials and Methods

The employed research method of the current paper is based on analysis of remaining pharmaceutical manuscripts of Persia during 9th to 18th century AD. These manuscripts are often spoken as Qarabadin books or traditional pharmacopeias describing the preparation procedures and considerations of compound medicines (9). In the current study, different dosage forms related to nasal application of herbal medicines and their therapeutic effects were derived from main Qarabadin books of Traditional Persian Medicine such as “Canon of Medicine” (written by Avicenna in 1025AD; includes 5 volumes that the second volume contains almost 800 natural medicaments which are mentioned monographically), book of “Qarabadin-e-azam” (a lithograph manuscript written by Hakim Azamkhan in 1853 AD; which has been organized based on pharmaceutical dosage forms in line with the diseases and body organs), “Qarabadin-e-ghaderi” (written by Ahmadshah Arzani in 1714 AD; which in that the pharmaceutical dosage forms have been differentiated from head to toe in line with disease types), “Qarabadin-e-kabir” (the largest pharmaceutical manuscript of Persian medicine written by Seyyed Mohammad Hossein Aghili Khorasani Shirazi in 1772 AD; which contains twenty chapters on pharmaceutical practice in the first part and followed by 28 parts on dosage forms), “Qarabadin-e-salehi” (written by Mohammad Saleh Ghaeni Heravi in 1766 AD; which is a lithograph manuscript in traditional pharmacy and encompasses almost 200 various pharmaceutical dosage forms along with preparation methods and related diseases), and Tohfat-ol-Moemenin (14-18). Since herbs were also applied individually in a form of nasal application, information on these medicaments was additionally derived using the latest and largest medical pharmacopeia of Persian medicine, Makhzan-ol-advieh (written by Seyyed Mohammad Hossein Aghili Alavi Khorasani Shirazi in 18th A.D; which is the largest and one of the latest traditional Persian pharmacopeias containing 28 chapters on natural medicines in alphabetical order involving 1698 monographs) (19). Selected manuscripts are extensively used as references for the Iranian PhD program in traditional pharmacy (20). 

The scientific names of the medicinal plants applied in formulations were then authenticated by use of botanical textbooks such as “Dictionary of Medicinal Plants” and “Popular Medicinal Plants of Iran” as well as “Pharmacographia Indica” (21-23). 

## 4. Results

Early Persian practitioners were aware of the systemic effect of nasally applied medicaments. Having the remained knowledge of other medical systems and their own experiences, they formulated many types of nasal dosage forms involving powder, solution and also gaseous preparations. The target site was either in the nasal area or even in the upper regions. But most of them were related to CNS disorders (19). Consistent with this belief, eleven types of nasal approaches encompassing powder, liquid and gaseous forms have been recorded in related manuscripts. Among them, powdered forms were subdivided to nasal insufflation (Nofookh) and errhine or sternutator medicine (Otoos). On the other hand, solutions or liquids contained nasal drop (*Qotoor*) and nasal snuffing drop (*Saoot*) as well as liquid snuff formulation (*Noshoogh*). Finally the last reported nasal dosage forms found as gaseous preparations were as fumigations (*Bakhoor*), medicinal vapor bathes or boiled aqueous extracts (*Enkebab*) and inhalation forms (*Lakhlakheh*) as well as aroma agents (*Ghalieh*). Interestingly, smelling a herb for a period of time was also considered as a pharmacological approach (*Shomoom*) (15-17). 

The form of fumigation (*Bakhoor*) was prepared by simply burning the herb and introducing the resultant smoke to the nasal fossa. Like in contemporary medicine, vapor bath (*Enkebab*) was the inhalation of vapors resulted from the decoction of a certain medicinal herb in a liquid base. In the form of aroma agent (Ghalieh) the desired parts of the herb were to be dispersed in vinegar of rosewater and smelled subsequently(17). The use of cooked musk in this form was very popular in the management of fainted patient (15). Inhalation (*Lakhlakheh*) was mentioned as the smelling of odor rising from either liquid or solid medicine kept in a bottle. The preparation was to be inhaled nasally during a day (16). These forms may be adapted to what is accepted as aromatherapy in current terminology (24), the approach which is now recommended for many medical interventions (25). Nasal drops (*Qotoor*) were applied as solutions similar to those of today’s medicine. For the preparation of *Qotoor* medicaments should have been thoroughly dispersed in water, milk, vinegar or other specific plant juice for the reduction of particle size. Nasal drops were often applied for the complications occurred in the nasal cavity. On the other hand, nasal snuffing drops (*Saoot*) were mentioned as watery or oily drops to be snuffed as entering the nasal fossa. Apparently this form was the most often applicable nasal liquid dosage forms in TPM. Watery or oily base were the same as for *Qotoor* (18). The recent dosage form was widely applied for diseases such as headaches, paralysis, apoplexy, stroke and other CNS disorders (19). Finally, liquid snuff formulation (*Noshoogh*) was defined as an applied liquid nasal dosage form in which a watery preparation of a medicine is sucked into the nose by inhaling (17). This form was similar to the *Saoot* form unless the container was different (14). Tables 1 and 2 present some examples of simple and compound medicines used as nasal dosage forms respectively. 

**Table 1.** Examples of Recorded Simple Nasal Formulations in Persian Pharmaceutical Manuscripts

Dosage Form	Scientific Name	Traditional Name (15,19)	Part Use	Applications
**Errhine** ** (*Otoos*)**	*Nicotiana tabacum* L.	*Tanbakoo*	Leaves	Errhine
**Fumigations (*Bakhoor*)**	*Zygophyllum fabago* L.	*Asl*	Fruits (Burnt)	Facial palsy
**Inhalation (*Lakhlakheh*)**	*Saccharum officinarum* L.	*Faniz*	Stem (Crude)	Brain and heart tonic
**Insufflation (*Nofookh*)**	*Ruta graveolens* L.	*Sodab*	Leaves (Dry)	Epistaxis
**Insufflation (*Nofookh*)**	*Glaucium corniculatum* (L.) Curtis	*Shaghaiegh*	Flowers (Dry)	Epistaxis
**Nasal Drop (*Qotoor*)**	*Alhagi maurorum* Medik.	*Kharshotor (haj)*	Aerial part (Extract)	Headache
**Nasal Drop (*Qotoor*)**	*Ziziphus spina-christi* (L.) Willd.	Sedr (konar)	Leaves (Vinegar extracted)	Epistaxis, Pediatric seizures
**Nasal Snuffing Drop (*Saoot*)**	*Ferula persica* Willd.	*sakbinaj*	Exudate (In oil)	Headache
**Olfaction (*Shomoom*)**	*Malus pumila* Mill.	*Tofah (sib)*	Fruits (Crud)	Brain and heart tonic
**Snuff (*Noshoogh*)**	*Euphorbia resinifera *O. Berg	*Oforbiun*	Exudate (In oil)	Vertigo
**Vapor Bathes (*Enkebab*)**	*Teucrium montanum* L.	*Marmahooz*	Aerial part (Decoction)	Headache

**Table 2.** Examples of Recorded Compound Nasal Formulations in Persian Pharmaceutical Manuscripts

Dosage Form	Ingredients (Scientific Name/Traditional Name)	Part Use	Applications
**Errhine (*Otoos*) (14)**	*Aloe vera* (L.) Burm. f./*Sebr*	Exudate	Facial palsy
	*Brassica nigra* (L.) K. Koch /*Khardal*	Fruits	
	*Citrullus colocynthis *(L.) Schrad./*Hanzal*	Fruits	
	*Nigella sativa* L./*Shoneez*	Seeds	
	*Piper nigrum* L./*Felfel*	Fruits	
	*Ruta graveolens* L./*Sodab*	Leaves	
**Fumigation (*Bakhoor*) (17)**	*Foeniculumvulgare* Mill./*Holbeh*	Seeds	Common cold, Rhinitis
	*Matricaria chamomilla* L./*Baboonaj*	Aerial part	
	*Mentha pulegium* L./*Phoodanaj*	Leaves	
	*Origanum majorana* L./*Marzanjoosh*	Leaves	
**Inhalation (*Lakhlakheh*) (In Rose Oil) (16)**	*Thymbra capitata* (L.) Cav./*Hasha*	Leaves	Headache
	*Nigella sativa* L./*Shoneez*	Seeds	
**Insufflation (*Nofookh*) (14)**	*Santalum album *L./*Sandal-e-sefid*	Bark	Epilepsy
	*Coffea arabica* L./*(Bonn) Ghahveh*	Seeds	
**Nasal Snuffing drop (*Saoot*) (Water base) (18)**	*Piper nigrum* L./*Felfel*	Fruits	Epistaxis
	*Punica granatum* L./*Romman (Anar)*	Fruit Skin	
**Nasal Drop (*Qotoor*) (Water base) (16)**	*Terminalia chebula* Retz./*Halileh*	Fruits	Epistaxis
	*Cinnamomum camphora* (L.) J. Presl /*Kafoor*	Exudate	
**Snuff (*Noshoogh*) (17)**	*Papaver somniferum* L./*Khashkhash*	Exudate	Insomnia
	*Cucurbita pepo* L./*Kadoo*	Seeds	
**Vapor Bathes (*Enkebab*) (14)**	*Papaver somniferum* L./*Khashkhash*	Seeds	Common cold
	*Triticum aestivum* L./*Gandom*	Seeds	
	*Vitis vinifera* L./*Khal (serkeh)*	Vinegar	

As powdered nasal dosage forms, insufflation or snorting agent (Nofookh) was reported as the practice of inhaling a solid substance and errhine or sternutator agent (Otoos) was introduced as a medicament in form of a fine powder with the potency of sneezing promotion (14). Persian scholars believed in the reduction of particle size of agents for nasal insufflation. Similarly, current investigations revealed the fact that particles around 100 microns in size possess useful insufflation properties for nasal administration (26). This from is also known as a way of substance abuse (27, 28). Application of errhine form was for diseases such as rhinitis, common cold, headaches, seizure, facial paralysis and other related ailments related to the nervous system (19). According to other traditional systems of medicine, errhine therapy was a popular approach to manage some CNS disorders (29, 30). In current pharmaceutical knowledge, the concept of nasal delivery has gained in interest. As a novel route for administration, nasal delivery is a potentially alternative way for systemic bioavailability in parenteral restricted applications (31). Desirable penetration, rapid onset of action, absence of hepatic first pass effect and protecting from gastric break down as well as promising results for CNS drug delivery via the olfactory region are some attributed advantages of this application route (32-34). Moreover, drugs which have high molecular weight or the ones which are potentially biosensitive such as peptides, vaccines, proteins and so on are good candidates to be delivered through nasal route (35). 

## 5. Discussion

Hence, many formulations in form of solution and powder have been applied in nasal delivery approach (36). Other than the systemic approaches, it should be noted that nasal drug application is highly applicable for local aspects such as maintenance therapy of nasal allergy, nasal congestion, sinusitis and infections (37). Taken as a whole, medieval nasal dosage forms may have similarities to those of current pharmaceutics. Inhalation form and aroma agents from the medieval reports correspond to the aromatherapy field and on the other side people consider the medicinal vapor bath and fumigation in common ethnomedical and ethnopharmaceutical approaches (38). A number of nasal drops have been formulated in contemporary pharmacy that may be inspired by the ancient knowledge. Also many clinical investigations were carried out on the efficacy of herbal nasal drops in current medicine (39, 40). Solid nasal dosage forms with the aim of systemic purposes are of novel pharmaceutical dosage form (41), while in traditional records this form was fully accepted for systemic illnesses (15). 

Although for many years, drugs have been administered nasally for local effect on the mucosa, Persian pharmaceutical literatures presented the route for various diseases especially for CNS. According to the advantages of nasal drug application and considering the medical approaches mentioned in medieval manuscripts, it is very likely that in the future more medicinal agents will come in the market in the form of nasal formulation having systemic purposes. In this regard, medieval pharmaceutical standpoints and considerations on nasal dosage forms could still be an interesting subject of study. Therefore the current work can briefly show the pharmaceutical knowledge on nasal formulations in Traditional Persian Medicine and clarify a part of the history of pharmacy in Persia. 

## References

[1] Borchardt JK (2002). The Beginnings of Drug Therapy: Ancient Mesopotamian Medicine.. Drug News Perspect..

[2] Kasthuri KT, Radha R, Jayshree N, Austin A, Thirugnanasambantham P (2010). Standardization and In Vitro Anti Inflammatory Studies of A Poly Herbal Oil Formulation–Megni.. Res J Pharm Technol..

[3] Buerki RA, Higby GJ, Swarbrick J (2006). Dosage Forms and basic preparations: History.. Encyclopedia of Pharmaceutical Technology..

[4] Levy G (1964). Effect of Dosage Form on Drug Absorption a Frequent Variable in Clinical Pharmacology.. Arch Int Pharmacodyn Ther..

[5] Khaleghi Ghadiri M, Gorji A (2004). Natural remedies for impotence in medieval Persia.. Int J Impot Res..

[6] Zargaran A, Zarshenas MM, Hosseinkhani A, Mehdizadeh A (2012). Jawarish, a Persian traditional gastrointestinal dosage form.. Pharm Hist (Lond)..

[7] Zargaran A, Zarshenas MM, Mehdizadeh A, Mohagheghzadeh A (2012). Oxymel in medieval Persia.. Pharm Hist (Lond)..

[8] Ramezany F, Ardakani MR (2011). Ali ibn Hosein Ansari (1330-1404): a Persian pharmacist and his pharmacopoeia, Ekhtiyarat i Badi i.. J Med Biogr..

[9] Levy M (1999). Medical Formulary or Aqrabadhin of Al-Kindi..

[10] Dhuria SV, Hanson LR, Frey WH, 2nd (2010). Intranasal delivery to the central nervous system: mechanisms and experimental considerations.. J Pharm Sci..

[11] Alsarra IA, Hamed AY, Alanazi FK, El Maghraby GM, Jain JJ (2010). Vesicular Systems for Intranasal Drug Delivery.. Drug Delivery to the Central Nervous System..

[12] Ugwoke MI, Verbeke N, Kinget R (2001). The biopharmaceutical aspects of nasal mucoadhesive drug delivery.. J Pharm Pharmacol..

[13] Putheti RR, Patil MC, Obire O (2009). Nasal Drug delivery in Pharmaceutical and biotechnology: present and future.. Sci Technol..

[14] Heravi MG (1765). [Qarabadin-e-salehi]..

[15] Avicenna . (1988). [Canon of Medicine]..

[16] Arzani A (1861). [Qarabadin Ghaderi]..

[17] Shirazi SA (1855). [Qarabadin-e-Kabir]..

[18] Azam Khan Cheshti H (1809). [Qarabadin-Azam]..

[19] Shirazi M (1992). [Makhzan ol Advieh]..

[20] (2007). [Program Book of Ph.D Degree in Traditional Persian Pharmacy]..

[21] Soltani A (2004). [Dictionary of Medicinal Plants]..

[22] Amin G (2005). [Popular Medicinal Plants of Iran]..

[23] Dymook W, Warden CJH, Hooper D (1893). Pharmacographica Indica..

[24] Baraniuk JN, Merck SJ (2009). Neuroregulation of human nasal mucosa.. Ann N Y Acad Sci..

[25] Cooke B, Ernst E (2000). Aromatherapy: a systematic review.. Br J Gen Pract..

[26] De Ascentiis A, Bettini R, Caponetti G, Catellani PL, Peracchia MT, Santi P (1996). Delivery of nasal powders of beta-cyclodextrin by insufflation.. Pharm Res..

[27] Vardakou I, Pistos C, Spiliopoulou C (2011). Drugs for youth via Internet and the example of mephedrone.. Toxicol Lett..

[28] Hill S, Sikand H, Lee J (2007). A case report of seizure induced by bupropion nasal insufflation.. Prim Care Companion J Clin Psychiatry..

[29] Amruthesh S (2008). Dentistry and Ayurveda - IV: classification and management of common oral diseases.. Indian J Dent Res..

[30] Bhatted S, Shukla VD, Thakar A, Bhatt NN (2011). A study on Vasantika Vamana (therapeutic emesis in spring season) - A preventive measure for diseases of Kapha origin.. Ayu..

[31] Jadhav KR, Gambhire MN, Shaikh IM, Kadam VJ, Pisal SS (2007). Nasal drug delivery system-factors affecting and applications.. Current drug therapy..

[32] Illum L (2012). Nasal drug delivery - recent developments and future prospects.. J Control Release..

[33] Landis MS, Boyden T, Pegg S (2012). Nasal-to-CNS drug delivery: where are we now and where are we heading? An industrial perspective.. Ther Deliv..

[34] Illum L (2003). Nasal drug delivery--possibilities, problems and solutions.. J Control Release..

[35] Arora P, Sharma S, Garg S (2002). Permeability issues in nasal drug delivery.. Drug Discov Today..

[36] Bertram U, Bodmeier R (2006). In situ gelling, bioadhesive nasal inserts for extended drug delivery: in vitro characterization of a new nasal dosage form.. Eur J Pharm Sci..

[37] Schipper NG, Verhoef JC, Merkus FW (1991). The nasal mucociliary clearance: relevance to nasal drug delivery.. Pharm Res..

[38] Bhat RB, Jacobs TV (1995). Traditional herbal medicine in Transkei.. J Ethnopharmacol..

[39] Chui SH, Shek SL, Fong MY, Szeto YT, Chan K (2010). A panel study to evaluate quality of life assessments in patients suffering from allergic rhinitis after treatment with a Chinese herbal nasal drop.. Phytother Res..

[40] Chan AS, Cheung MC, Sze SL, Leung WW, Shi D (2011). An herbal nasal drop enhanced frontal and anterior cingulate cortex activity.. Evid Based Complement Alternat Med..

[41] Bhise SB, Yadav AV, Avachat AM, Malayandi R (2008). Bioavailability of intranasal drug delivery system.. Asian J Pharm..

